# More than Meets the Mind’s Eye? Preliminary Observations Hint at Heterogeneous Alpha Neuromarkers for Visual Attention

**DOI:** 10.3390/brainsci9110307

**Published:** 2019-11-02

**Authors:** Emmanuelle Tognoli

**Affiliations:** Center for Complex Systems and Brain Sciences, Florida Atlantic University, 777 Glades Road, Boca Raton, FL 33431, USA; tognoli@ccs.fau.edu

**Keywords:** EEG, alpha, xi, Posner, covert attention

## Abstract

With their salient power distribution and privileged timescale for cognition and behavior, brainwaves within the 10 Hz band are special in human waking electroencephalography (EEG). From the inception of electroencephalographic technology, the contribution of alpha rhythm to attention is well-known: Its amplitude increases when visual attention wanes or visual input is removed. However, alpha is not alone in the 10 Hz frequency band. A number of other 10 Hz neuromarkers have function and topography clearly distinct from alpha. In small pilot studies, an activity that we named xi was found over left centroparietal scalp regions when subjects held their attention to spatially peripheral locations while maintaining their gaze centrally (“looking from the corner of the eyes”). I outline several potential functions for xi as a putative neuromarker of covert attention distinct from alpha. I review methodological aids to test and validate their functional role. They emphasize high spectral resolution, sufficient spatial resolution to provide topographical separation, and an acute attention to dynamics that caters to neuromarkers’ transiency.

## 1. Introduction

A crucial set of experiments by Michael Posner and colleagues [1] buttressed the theory that minds are equipped with a covert visual orienting system to enhance detection of targets even as they lie out of the foveal center of the participant’s overt visual attention. This is the notorious spotlight that had been postulated by James in 1890 [2], and evidenced by Helmoltz [3]—see also the reviews and controversies [4,5,6,7,8] and overviews of neuroanatomical bases [9,10,11] that lent credit to a view of multiple independent networks and processes for attention [9]. 

In a separate stream of research founded by Hans Berger in the 1920s [12,13], a rhythmic brain wave at about 10 Hz was shown to transpire from the scalp of human participants and reacted to such events as eye opening, involuntary attention to sudden startle from gunshot sound, other auditory, visual, olfactive, tactile, and pain stimuli, voluntary concentration, anesthesia, medications, and a variety of clinical conditions [12,13,14,15,16,17]. This brain wave is a dominant activity in human waking electroencephalogram (EEG) [18]. After the controversy of its cerebral origin was finally settled [15], what came to be known as the alpha wave [14] rose as one of the most studied electrophysiological phenomenon, with firmly established correlation to attentive processes [19,20,21,22]. 

Here, we restrict the name alpha to the 10 Hz phenomenon peaking in parieto-occipital regions when eyes are closed or attention and vigilance reduced. Alpha’s anticorrelation with attentive behavior was noted from the start [12,13,14] and continued to be observed [23] after warning signals of target occurrence [24] and in relation with many tasks derived from Posner’s cueing paradigm, consensually with a contralateral organization, that is, increase power opposite the stimulus side or decrease power ipsilateral to it [25,26,27,28,29,30,31,32,33,34,35,36,37,38] after target onset as well as during the cue period [26,39] and even in the absence of ultimate target [40]. Alpha is also modulated by temporal expectations [33] and abides to reverse causal inference that finds more omissions when background alpha is intrinsically larger [28,41], externally entrained [42] or perturbed [11]. 

The functional complexity of 10 Hz oscillations in posterior regions has also been noted [43,44]. First, a so-called paradoxical alpha response (increasing alpha during some exemplars of attentive behavior in violation of the otherwise strong record of alpha suppression during attention, see above) has led to a fervent debate [19,45,46,47,48] that appears to have been resolved with a distinction between endogenous and exogenous attention [21,46]: Internally-generated attentional processes are purported to increase alpha to actively inhibit sensory information. This hypothesis retained the idea of a unitary alpha that equally suppresses exogenous and endogenous distractors, but firm evidence of complete anatomical and dynamical equivalence remains unfulfilled. Second, studies of oscillatory power that paid close attention to the timing of oscillatory processes have suggested a complex temporal organization of posterior 10 Hz activities with different sub-bands of alpha playing a role at different moments [18,24,49,50,51], see also [31]. These studies beg for finer-grained models for the relation between local rhythmic activity and attentional processes. Finally, several studies of functional connectivity also suggest a complex spatiotemporal organization [11,27,52,53,54].

**Figure 1:**
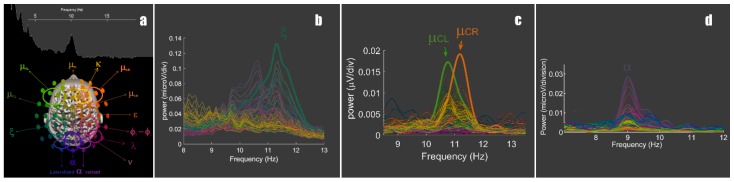
The 10 Hz frequency band carries a number of regionally and functionally specific neuromarkers: (**a**) An overview of their peak scalp locations is provided over a colorimetrically-encoded electrode map (recalling that the inverse problem prevents direct cortical localization); (**b**) xi is found in the upper 10 Hz band, with maxima at left centroparietal scalp locations as shown by the green color of its peak inherited from the colorimetric mapping; **(c)** an exemplar pair of Rolandic mu (here denoted mu central left and right) illustrates the limited spatial overlap that left mu has with xi, though both share the upper 10 Hz band; **(d)** an exemplar of alpha rhythm shows discrepancy both in spatial and spectral organization, with alpha having a slower peak frequency (population mode robustly at 10 Hz [57,58]) and a spatial distance to xi of several centimeters, as manifested with the distinct color (inherited from spatial location and not randomly assigned, see (**a**) for legend). Spectra from (**b**), (**c**), and (**d**) are sampled from different subjects and tasks.

In the following, I introduce a case study from a small-scale pilot experiment that asked subjects to sustain the dissociation between fixation and covert attention (“looking from the corner of the eyes”). During this task, a 10 Hz neural activity was uncovered that is clearly distinct from alpha (Figure 1b), and I used its existence to develop the hypothesis on a multifaceted model of 10 Hz rhythms’ contribution to attentional processes. I based this proposed model on a discrete view of neural oscillations that are spatially specific (e.g., [55,56] and Figure 1a), appearing intermittently over time [57]. This view was gained from a large improvement in spectral and spatial resolution of EEG analysis, and I outline the methodological requirements that might allow to further study the interplay of 10 Hz rhythms, alpha, and others, in attentional processes. 

## 2. Materials and Methods

A subject participated in multiple sessions of an EEG recording (protocol approved by Florida Atlantic University’s institutional review board; and written informed consent obtained prior to the experiment). The subject was a right-handed male, healthy young adult (early 20s), with no history of neurological disease, and with normal vision and audition. The sessions were aimed at developing a brain–computer interface [59] and consisted of various ideomotor activities such as executing or imagining movements of the mouth, knees, feet, hands, and fingers by self or other, and lifting of small objects. To control for the potential confound of spatially shifted attention, especially in the case of imagined and executed lower limb movements, a small task was added to the protocol asking the subject to preserve fixation on a crosshair in the center of the computer screen while looking from the corner of the eyes at an unmarked location at the top or bottom of the screen (alternations of 15 s epochs looking from the corner of the eyes and 5 s epochs releasing attention to the central fixation, cumulative duration, 120 s for each direction). This task was created under the rationale that subjects would comply to fixation (validation with EOG) yet might covertly orient their attention to the spatial locus where the targeted body part lay. Since there were no explicit state variables to detect and control for such covert occurrence, we reasoned that sample neuromarkers for this covert activity were important to collect and characterize spatiotemporally. The experiments were conducted in a sound-proof electromagnetically shielded chamber. EEG was recorded using a 60-channel EEG cap with Ag–AgCl electrodes (Falk Minow Services, Herrsching, Germany) arranged according to the 10 percent system [60] (including midline and rows 1 to 8). Electrodes were laid on standard elastic caps whose positioning emphasized the accuracy of vertex electrodes (midway between nasion and inion) and the adequacy of the midline to improve interpretations of lateral symmetries. Electrode impedances were maintained below 10 kΩ, and special attention was paid to the reference electrodes, a pair of digitally linked mastoids (subjected to removal of lipidic film with alcohol swab, double abrasion with hair brush and then gel nuprep, careful adhesive, and compressive securing of the electrodes with tape and elastic cap), leading to their impedance to be low and matched [61]. The ground electrode was located at electrode FPz. The signals were fed to an amplifier (Synamp2, Neuroscan, Texas). The signals were analog-filtered (Butterworth, bandpass from 0.05 Hz (−12 dB/octave) to 100 Hz (−24 dB/octave-)), amplified (gain of 2010), and digitized at 1000 Hz with a 24-bit ADC in the range 0–900 microV (vertical resolution of 0.11 nV). Electro-oculographic (EOG) traces were obtained from two pairs of electrodes placed above and below the right eye (vertical EOG) and on the canthus of each eye (horizontal EOG) to ascertain compliance with instructions not to move the eyes during the tasks. 

Three EEG analysis strategies are succinctly presented below, which have been described elsewhere. Multielectrode spectra are obtained via the fast Fourier transform on epochs prepared for enhanced spectral resolution, that is, with the sampling of a longer time interval (e.g., 8.192 or 16.384 s at sampling rate = 1 k Hz) that provides a bin size of 0.06 to 0.12 Hz, much more detailed than a usual 1 Hz resolution and therefore amenable to detecting small discrepancies in frequency of the brain’s many 10 Hz activities (Figure 1, see also [55]; strategies to achieve same resolution with smaller epochs can be found in [56]). The spatiotemporal analysis of band-passed filtered EEG uses gently tuned Butterworth filters chosen for their flat passband and applied in both time-positive and time-negative directions to prevent phase distortion [56]. Envelopes are used to scrutinize instantaneous power changes. They were obtained after mean-removed bandpass-filtered signals were rectified and smoothed with a moving average of 100 milliseconds. 

## 3. Results

**Figure 2:**
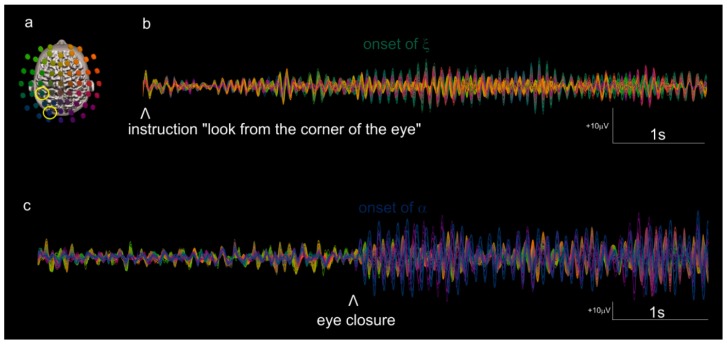
The transition to xi activity during covert shifts of attention is compared to the onset of alpha during closure of the eyes: (**a**) shows a colorimetric map with two electrodes of interest, CP3 (xi, electrode colored green) and PO3 (lateralized variant left alpha, electrode colored blue, see [63]), underlined by yellow circles. (**b**) shows a sample task during which a subject held his attention at the top of the computer screen while fixating the screen’s center (i.e., fovea centrally located, and attentional spotlight maintained several degrees away vertically, verified by the absence of saccades or eye movement in EOG traces). Note the onset of ample waves with green color, about 1.5 s after instruction, and sustained for seconds. For comparison, (**c**) shows the 10 Hz band during quiet rest with eye open (left of the marker) and with eye closed (right) in another subject. Note the characteristic low amplitude, moment-to-moment variability in spatiotemporal patterns (color change) prior to eye closure, and the large amplitude activity in posterior locations (colored blue and magenta) afterwards. (**b**) is filtered in the 10–13 Hz band, (**c**) in the 8–12 Hz, in agreement with each target activity’s spectral distribution.

Figure 2b shows a sample of bandpass-filtered EEG activity collected after on-screen instructions required the subject to covertly move his/her attention to a peripheral location. Xi was observed during covert attention tasks (up and down), execution and imagination of leg movements, imagination of someone else’s lifting and releasing a small object, and imagination of self-releasing a grasped object. It was absent in the other ideomotor tasks for this study (i.e., no specific departure from background spectral distribution). Note that xi had not been previously observed in studies of rest with eyes opened or closed, or in a variety of sensorimotor and social coordination tasks (e.g., [56]). The green brainwave that appeared during the covert attention tasks is uncharacteristic of waking EEG in two respects: It has an unusually sustained duration that is infrequently observed during mental activities (compare with, e.g., rest eye opened, with its fast succession of spatiotemporal patterns, see also [56,57,62] for reference dynamics generally lasting one or two cycles). Further, its topography remains several centimeters away from the alpha rhythms. For comparison, Figure 2c provides the dynamics of alpha during an eye opened and eye closed task in a different subject. Note the ample oscillations dominated by blue and magenta color that become sustained and ample at eye closure.

**Figure 3:**
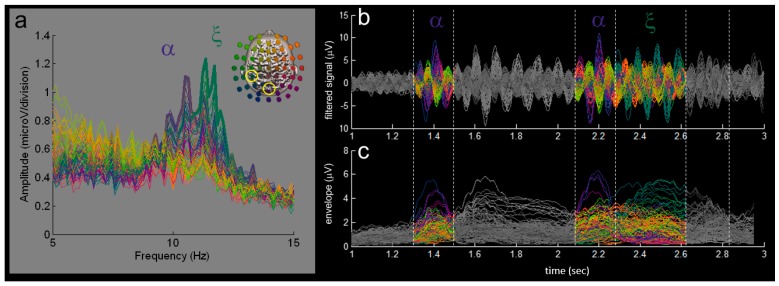
Xi and Alpha differ spectrally and temporally. (**a**) shows a colorimetric spectrum during covert attentional shift upward. Note that in this single subject, the peak of alpha at 10.62 Hz differs from the peak of xi at 11.29 Hz. (**b**) shows a sample bandpass filtered EEG in the 8–13 Hz. Alpha and xi patterns are outlined (other patterns obscured for simplicity). Note that the patterns are not co-occurring in time, as if their functional processes excluded each other. To aid segmentation, signal envelope is plotted in (**c**).

The spatial discrepancy between xi and alpha cannot be attributed to irregular electrode positioning or idiosyncratic orientation of the alpha generators shifting the forward projection of alpha generators on the scalp: Analysis within subject confirms that they are two distinct phenomena. They were found to co-occur at the rough timescale of entire tasks, for instance, during covert shift of attention upward (Figure 3a), and they differed in both spatial and spectral distribution as shown by their different color and peak frequencies. They also exhibited distinct temporal organization: Although some alpha patterns were intermittently present in the tasks where xi was discovered, alpha and xi occurred at different times (Figure 3b,c).

## 4. Discussion

In a pilot study manipulating the location of the attentional spotlight away from fovea, we found clear evidence of a 10 Hz activity distinct from alpha in a subject [59]. The dissociation was supported by differences in spectral, spatial, and temporal organization (Figure 1, Figure 2 and Figure 3), and we chose to name the newly characterized neural activity at electrode CP3 ‘xi’. Naming neuromarkers is instrumental in beginning to confront and document their anatomofunctional specificities [55,56] and to clarify a literature record that oftentimes accretes unrelated oscillatory phenomena (see also the spectacularly detailed insights from [43] along the same lines). In keeping with this logic, like many others, we found alpha to react to subjects’ drowsiness (more alpha at the end of experimental sessions than at the beginning), amount of visual stimulation (quantity of movement seen, brightness), and subjective self-report of task engagements (not shown). By contrast, xi reacted to instructions to look from the corner of the eyes, and to tasks where covert attending to distal body parts is implicitly assumed. Alpha was of course increased by inattention; however, inasmuch as it was driven by our instruction to covertly attend to peripheral location, xi was increased, not decreased, by attention. We therefore raise the possibility that alpha and xi are two independent attentional neuromarkers within the 10 Hz band, contributing in unique ways to the functional architecture outlined in the introduction [9,10,64].

In covert tasks such as the above, there are limited tools to cross-validate the subject’s mental activity and the temporal footprint of their occurrence. Compliance to fixation was verified from EOG, and a brain–computer interface using xi was successfully developed [59], suggesting that our instructions were coherently understandable in the training and test phases of such research. In hypothesizing on the mental activity associated with xi, I ought to raise three possibilities: (1) xi might represent an idiosyncratic activity that is not shared in the general population and its exposition will be limited to the present report; (2) xi might represent the neural mechanisms that shift the attentional spotlight away from the foveal center; and (3) xi might represent the processes that maintain the dissociation between overt and covert attention, perhaps via oculomotor control areas to repress saccades. In that respect, discussions with a colleague raised the issue of xi’s spatial and functional proximity to left Rolandic mu rhythm, a well-known sensorimotor activity, which remains to be more fully studied (although our specimen of xi were more posterior than most left mus on record with the montages used in my laboratory). Rolandic mu rhythms could not be distinguished from background EEG after and during the movement execution tasks, preventing us from making definitive assessments of the similarity between xi and a left mu rhythm.

In the task that was set up, subjects had 15 s to best sustain a covert shift of attention. This prolonged duration (initially designed to gain spectra resolution) likely helped to obtain a robust signal (provided subjects’ abilities to sustain the mental effort this long). It is possible that xi activity is also buried in prior cueing tasks, but too brief to rise over the background of a generally dominant alpha activity in human waking EEG. In that respect, I have introduced some sensitive but time-consuming tools to achieve a detailed analysis of spatiotemporal dynamics of the EEG and to uncover crucial activities that are obscured by an unfavorable mixture of small amplitude and short duration: starting with spectra computed with increased spectral resolution, it is possible to distinguish closely-spaced spatiospectral activities (e.g., Figure 3a) and avoid a confound where discretely distinct neuromarkers appear as unitary processes with subtle spatial shift in serial mapping of frequencies with coarse spectral resolution. The study of spatiospectral organization can be followed with the examination of spatiotemporal organization of bandpass-filtered EEG (Figure 2b,c and Figure 3b), its envelope (Figure 3c), and in more details, the phase organization of its oscillations [56,62]. Those painstaking studies allow forming hypotheses on the nature, sources and coordination dynamics of neural oscillations and are aimed to precede robust hypothesis-confirming investigations.

## 5. Conclusions 

In conclusion, this case report of a spatial, spectral, and functional dissociation between alpha and xi supplies the foundation to further experiments, with the potential outcome to break the unitary view of alpha rhythm and attentional processes, which many other electrophysiologists have already suggested [24,31,35,37,54,65].
